# Curious Case of Midwifery

**Published:** 1880-04

**Authors:** R. A. Kinloch

**Affiliations:** Charleston, S. C.


### CURIOUS CASE OF MIDWIFERY.

BY R. A. KINLOCH, M.D., CHARLESTON, S. C.

On Wednesday, November 19th, at 11 A.M., I visited Lela P., a finely developed and robust mulatto, aged 27 years, in labor with her third child. She had been in pain for several hours. Upon examination, I found the labor but little advanced; os open the size of a quarter of a dollar; head presenting; tissues soft, relaxed, and promising an early termination of the confinement. The pains were infrequent and not strong; they increased gradually during the day, and at 9½ P.M. the waters were discharged. After this, all pain subsided and patient slept the entire night.

On Thursday morning, 10 A.M., found os more open, but pains so feeble and transient that I thought it advisable to relieve the uterine inertia. I prescribed 20 drops of the fluid extract of ergot every hour, until five doses were taken, and requested that I should be sent for when the pains became active. The summons came for me late in the evening. Found patient restless, with frequent and severe pains, but not of the "bearing down" kind. Examination, discovered a kind of polypoid growth occupying the upper part of the vagina, which, upon first touch, felt more like the genitals of a male fœtus than anything else. I was at first startled at the idea of a breech presentation having been substituted for what certainly was a head case in the morning. Further investigation, however, very soon led me to abandon this view of the case; but I continued puzzled in regard to the real nature of the presenting tumor. I could easily circumscribe this with my finger within the external os. It seemed as large as an egg, though flaccid, and as strikingly pediculated as a common polyp coming from the cervix. No internal os could be made out, though the stem of the tumor passed up to where this opening was supposed to be located. Outside of the patulous external os, through the tissues forming the vault of the vagina, could be felt what I took to be the presenting head. Believing that the inner os was tightly encircling the tumor, I made repeated efforts to get my finger within this to demonstrate the fact, and to learn more of the real nature of the case. I entirely failed. The elongated polypoid character of the growth suggested the possible existence of an *encephalocele* or a *meningocele* that had passed through the inner os and been somewhat changed by its tight and sharply cut margins.

The restlessness and suffering of my patient called for relief, and requiring assistance, in any attempt at delivery, I sent for my instruments, and at the same time requested the aid of my friend, Prof. F. M. Robertson, who had had fifty years experience, and had long been a teacher of obstetrics. Upon the doctor's arrival, without giving him any information, I begged him to examine the case and give me his opinion of the presentation. He did so, carefully and long, and then turning to me, said he had never before encountered such a condition. He could make out the head through the vaginal vault, but he could not affirm anything as to the tumor, nor could he find the inner os. We finally determined to open up the case by puncturing the head through the presenting tumor, as a first step towards attempting delivery. The fœtal heart could not be heard, and we concluded that the child was dead. The patient had been taking chloroform in small quantities, but had never been brought thoroughly under its influence. Anæsthesia was now fully induced, and I proceeded to make another and final examination previous to using the *vectis*. This effort suddenly disclosed to me the inner os. Pressing my finger closely around the stem of the tumor, it suddenly slipped higher up, entered the os, now relaxed by the chloroform, and impinged fairly against the resisting head. A second finger, and then a third, and a fourth, was made to follow. The os yielded more and more under the force of this entering wedge, and I was soon enabled to clear up all doubts, and to recognize the fact that the tumor was only a remarkably developed "*caput su'cedaneum.*" Never before had either of us seen a "*cuput*" so shaped, or so constructed, nor could we have before believed that the tissues of the s·alp were capable of such œdematous elongation or development.

My success so far now induced me to change my plan of proceeding. I put aside the *vectis*, and in due time adjusted Hodge's long forceps to the head. Craniotomy, even with a dead fœtus, is horrible to contemplate, and I preferred risking the use of the forceps, even with an os rigid enough to make me fear a laceration. By slow and careful traction the delivery of a dead female child was soon effected. Examination after the removal of the placenta revealed the fact that the cervix had sustained a rent about an inch long upon the left side, passing through the margin of the external os.

Patient reacted well; had no serious hemorrhage, and was left after an hour in a very comfortable condition. She did well for six days. Used anti-septic injections to vagina during this period. Upon the seventh day I had occasion to leave the city. On the morning following, patient was suddenly prostrated to syncope by a copious bleeding. Brandy was given freely until assistance could be had. Then, under professional skill, the vagina was tamponed with styptic cotton. No further hemorrhage ensued. The tampon was removed after two days. Patient recovered in good time, and has since enjoyed excellent health.

*Remarks.*—It may be interesting for me to furnish some details of the previous history of this woman. She had married at the age of 21, was of good constitution, but suffered from painful menstruation, and never conceived until after having been married two years. I then attended her for the first time and divided the *cervix* by the bilateral incisions; also dilated with laminaria tents at times during a period of several months. She conceived nine months after my first operation, and was delivered by me, with forceps, of the first child, a well-developed male. I also attended her in her second confinement, more than a year after her first. This second child, a male, was still-born, after a tedious labor of nearly three days. I had not been summoned at the later stage of the labor, but upon accidentally visiting the patient found the child had long been arrested at the vaginal outlet. It only slowly emerged after my making forcible pressure over the uterus. The history of the third labor is that given above.

There are, undoubtedly, several points of interest pertaining to this history and case: 1. We may inquire into the causes operating to produce a "*caput succedaneum*" so unlike in shape and developments those we usually meet with. 2. The probable influence of the original operation upon the *cervix* in determining the condition of the os at the time of labor. 3. The probable *role* enacted by the ergot administered. 4. The great value of chloroform in opening an os which was practically impenetrable. 5. The source and the cause of the bleeding seven days after delivery.

In regard to the first point, we may remark that the "*caput succedaneum*," as usually observed, is found in connection with a well dilated os, and towards the termination of labor, when the pains are most expulsive in character, and the head in the axis of the vaginal canal. The œdema of the scalp which occasions the formation of the tumor, is brought about by the want of support which the tissues experience in the vaginal canal as the contracting uterus is forcing its contents downwards. The active constriction of the os itself has but little to do with this. Hence these tumors of the fœtal head are not pediculated, but rather conical or roundish, with broad base in contact with the skull.

In the case before us, the circle of the inner os was small, and actively constricted the scalp tissues as these were forced through it and into the vaginal canal. The tumor resembled and grew like a hernial tumor does as its circulation becomes interfered with; but the constriction at the opening in this case was more active than is ever possible in connection with a hernial ring. The constriction was likewise passive in virtue of the mere rigidity of the margins of the os.

This later condition was more assured, perhaps, by the effect of my original operation upon the cervix. I am inclined to believe that the cicatricial tissue which much result from such operations (it could not be felt in my examination at the time of labor) favors the rigidity of the cervix and the *os*, and thus furnishes an additional impediment to natural labor. At the same time, I am glad to be able to say, from experience in a few cases, that these operations on the cervix offer no formidable complication. I have known instances where conception, after such operations, has been followed by natural and easy delivery. Statistics upon this subject would be valuable.

Third. I am inclined to attach much importance to the action of the ergot in the case. Before the administration of the drug, the cervix was soft, and the os apparently very dilatable. It is not my habit to give ergot in labor, but in this instance, at the particular time noted, it seemed really to be indicated. I am now disposed to think that the use of the agent was injudicious and hurtful.

The active part played by the circular fibres of the uterus, or the inner os, (be it remembered that the outer os was soft and patulous) was most assuredly due to the ergot. Had this agent been longer continued, or the labor not been terminated by art, we might have reasonably looked for rupture of the uterus at some point. The administration of large doses of chloral hydrate, together with the ergot, would, perhaps, have been better practice, and have worked out different results.

Fourth. The value of chloroform in a rigid os has long been recognized, and can be safely given, however, only while the physician is present. It did not seem to be indicated when the ergot was prescribed. Chloral hydrate has the advantage over chloroform that it can be given at stated times, regardless of the presence of the practioner, and, moreover, it does not seem to retard the labor, as chloroform most assuredly will do at certain stages of its progress.

While we fully understand the value of chloroform as a relaxant, we were not prepared for the very marvelous promptness with which it acted. Without it we feel convinced we could not have opened the os sufficient to inform ourselves of the true nature of the case. And yet, with it, in a few minutes, our hand and our instrument were passed through an opening that we had in vain endeavored to recognize.

Fifth. The hemorrhage on the seventh day we can attribute in no way to the uterine cavity. There were no placental remains to escape, and the contraction and involution had gone on securely. It is most probable that the bleeding resulted from the separation of a slough at the point of rupture in the cervix. It was really secondary and traumatic. The tampon carried well up to the cervix did successful work. The fissure may give trouble in the future, but so far it has not developed any symptom. As we are not one of those who hunt up such abnormities simply to remedy them, we shall not at present interfere.

